# Pulmonary blood volume index as a quantitative biomarker of haemodynamic congestion in hypertrophic cardiomyopathy

**DOI:** 10.1093/ehjci/jez213

**Published:** 2019-08-29

**Authors:** Fabrizio Ricci, Nay Aung, Ross Thomson, Redha Boubertakh, Claudia Camaioni, Sara Doimo, Mihir M Sanghvi, Kenneth Fung, Mohammed Y Khanji, Aaron Lee, James Malcolmson, Cesare Mantini, José Paiva, Sabina Gallina, Artur Fedorowski, Saidi A Mohiddin, Giovanni Donato Aquaro, Steffen E Petersen

**Affiliations:** 1 Institute for Advanced Biomedical Technologies, Department of Neuroscience, Imaging and Clinical Sciences, “G.d'Annunzio” University, Via Luigi Polacchi, 11 - 66100 Chieti, Italy; 2 William Harvey Research Institute, NIHR Barts Biomedical Research Centre, Queen Mary University of London, Charterhouse Square, London EC1M 6BQ, UK; 3 Barts Heart Centre, St Bartholomew's Hospital, Barts Health NHS Trust, West Smithfield, London EC1A 7BE, UK; 4 Department of Clinical Sciences, Lund University, Skåne University Hospital, SE-205 02 Malmö, Sweden; 5 Fondazione Villa Serena per la Ricerca, Viale Leonardo Petruzzi, 42 - 65013 Città Sant'Angelo, Italy; 6 Cardiovascular Department, Azienda Sanitaria Universitaria Integrata, University of Trieste, via Pietro Valdoni, 7 - 34149 Trieste, Italy; 7 MRI Lab, Fondazione Toscana G. Monasterio, via G. Moruzzi 1, 56124 Pisa, Italy

**Keywords:** pulmonary blood volume, diastolic dysfunction, hypertrophic cardiomyopathy, cardiovascular magnetic resonance, echocardiography

## Abstract

**Aims:**

The non-invasive assessment of left ventricular (LV) diastolic function and filling pressure in hypertrophic cardiomyopathy (HCM) is still an open issue. Pulmonary blood volume index (PBVI) by cardiovascular magnetic resonance (CMR) has been proposed as a quantitative biomarker of haemodynamic congestion. We aimed to assess the diagnostic accuracy of PBVI for left atrial pressure (LAP) estimation in patients with HCM.

**Methods and results:**

We retrospectively identified 69 consecutive HCM outpatients (age 58 ± 11 years; 83% men) who underwent both transthoracic echocardiography (TTE) and CMR. Guideline-based detection of LV diastolic dysfunction was assessed by TTE, blinded to CMR results. PBVI was calculated as the product of right ventricular stroke volume index and the number of cardiac cycles for a bolus of gadolinium to pass through the pulmonary circulation as assessed by first-pass perfusion imaging. Compared to patients with normal LAP, patients with increased LAP showed significantly larger PBVI (463 ± 127 vs. 310 ± 86 mL/m^2^, *P* < 0.001). PBVI increased progressively with worsening New York Heart Association functional class and echocardiographic stages of diastolic dysfunction (*P* < 0.001 for both). At the best cut-off point of 413 mL/m^2^, PBVI yielded good diagnostic accuracy for the diagnosis of LV diastolic dysfunction with increased LAP [C-statistic = 0.83; 95% confidence interval (CI): 0.73–0.94]. At multivariable logistic regression analysis, PBVI was an independent predictor of increased LAP (odds ratio per 10% increase: 1.97, 95% CI: 1.06–3.68; *P* = 0.03).

**Conclusion:**

PBVI is a promising CMR application for assessment of diastolic function and LAP in patients with HCM and may serve as a quantitative marker for detection, grading, and monitoring of haemodynamic congestion.

## Introduction

Hypertrophic cardiomyopathy (HCM) is the most common inherited heart muscle disorder and an important cause of heart failure (HF) with preserved left ventricular (LV) ejection fraction.^1^^,^^2^ Diastolic dysfunction is a major contributor to the pathophysiology of HF in HCM, which encompasses a complex sequence of interrelated mechanisms including LV hypertrophy, intraventricular obstruction, microvascular ischaemia, and myocardial fibrosis.^3^ Evidence of diastolic dysfunction is frequently observed in HCM^4^ and is known to have a major impact on symptom severity, functional capacity, medical treatment, and prognosis.^5–7^

Echocardiography is the non-invasive modality of choice for the semi-quantitative evaluation of LV diastolic function. Using echocardiography, a comprehensive multi-parameter algorithm is recommended for the estimation of elevated mean left atrial pressure (LAP).^8^^,^^9^

Despite this, the identification of accurate and reproducible indices for quantitative assessment of haemodynamic congestion in HCM is still an unmet clinical need, in particular, to allow for early detection of disease progression and treatment guidance. An alternative approach to assess the presence and severity of diastolic dysfunction would be by measuring to what extent the elevated LV filling pressure is transmitted retrogradely into the pulmonary circulation leading to an increase in central transit time,^10^ pulmonary blood volume,^11^ and eventually to increased pulmonary capillary hydrostatic pressure (haemodynamic congestion). Pulmonary blood volume index (PBVI) by first-pass perfusion cardiovascular magnetic resonance (CMR) imaging has been shown to differentiate between stages of diastolic dysfunction in patients with HF and reduced LV ejection fraction and has been proposed as a quantitative marker of HF useful for quantification and monitoring of haemodynamic congestion.^11^^,^^12^ In this study, we aimed to assess the diagnostic accuracy of PBVI by CMR for the detection and grading of haemodynamic congestion in patients with HCM and preserved LV ejection fraction.

## Methods

### Study design and population

This is a retrospective study of consecutive patients recruited into the Barts BioResource medical research programme complying with the 1975 Declaration of Helsinki and approved by our Institutional Ethics Committee. The Barts BioResource is a joint partnership between Barts Health NHS Trust, the William Harvey Research Institute/Queen Mary University of London, and University College London with support from the National Institute for Health Research Barts Biomedical Research Centre aimed to establish a research repository of consented patients clinically managed by Cardiac Services at Barts Health NHS Trust. All patients gave written informed consent and received standard optimal medical therapy according to the current guidelines. We identified all consecutive HCM outpatients undergoing CMR stress perfusion imaging for clinical purposes between January 2015 and September 2017.

Exclusion criteria were as follows: (i) age <18 years; (ii) presence of any contra-indication to CMR and/or to administration of gadolinium-based contrast agents; (iii) atrial fibrillation or frequent ventricular ectopy during image acquisition; (iv) any hospitalization, worsening of the functional status, changes in medical treatment occurred in the time interval between transthoracic echocardiography (TTE) and CMR; (v) at least moderate tricuspid valve regurgitation by TTE; (vi) known significant coronary artery disease; and (vii) LV ejection fraction <50%.

All patients underwent a standard clinical assessment including medical history, physical examination, routine blood tests, transthoracic echocardiogram, and contrast-enhanced CMR.

### Transthoracic echocardiography

All patients underwent both standard TTE (Vivid E9^TM^, General Electric) and CMR examinations [median time interval 5 days, interquartile range (IQR) 3–10]. A comprehensive echocardiographic assessment of LV diastolic function was performed in agreement with the current international guidelines,^8^ including structural assessment of LV size and mass, left atrial (LA) volume, mitral inflow and pulmonary venous flow patterns, pulsed-wave tissue-Doppler imaging of mitral annular velocities, peak velocity of tricuspid regurgitation, and systolic pulmonary artery pressure (sPAP). LV diastolic dysfunction with increased LA pressures was defined as the presence of a Grade II or III diastolic dysfunction by Doppler echocardiography, as described previously.^8^

### Cardiovascular magnetic resonance

All patients were examined on two Magnetom Aera 1.5 T scanners (Siemens Healthineers, Erlangen, Germany) using an 18-channel cardiac phased array anterior coil in combination with the spine coil (up to 12-channel coil elements). Biventricular function was assessed by breath-hold, steady-state free precession (SSFP) cine short-axis imaging covering both ventricles. Typical scan parameters were: field of view 360 × 290 mm^2^, 8 mm slice thickness with a 2 mm inter-slice gap, repetition time 2.7 ms, echo time 1.14 ms, flip angle 80°, acquisition matrix 208 × 169, reconstruction pixel size 1.73 × 1.73 mm^2^, acquired temporal resolution 35.1 ms (13 views per k-space segment), and 30 calculated cardiac phases. CMR perfusion imaging was performed with the first-pass technique as described previously.^11^ Briefly, a bolus of gadolinium-based contrast agent (Dotarem, Guerbet, France, 0.05 mmol/kg at an injection rate of 4 mL/s) was injected into a peripheral vein, followed by a saline flush (injection rate 4 mL/s). Adenosine was given intravenously at 140 μg/kg/min for 4 min. First-pass images were obtained using cardiac-triggered gradient echo-train SSFP pulse sequences. Images from three short-axis planes (basal, middle, and apical) were acquired with the following parameters: typical field of view 360 × 270 mm^2^, slice thickness 8 mm, slice gap 12 mm, repetition time 2.5 ms, echo time 1.04 ms, flip angle 50°, bandwidth 1085 Hz/pixel, 60 dynamics, acquisition matrix 111 × 192, and a parallel acceleration factor *R* = 3 (TGRAPPA).^13^ Image acquisition was begun at the same time as the contrast media injection.

### Image analysis

All CMR studies were analysed off-line using cvi^42^ post-processing software (Version 5.1.1, Circle Cardiovascular Imaging Inc., Calgary, Canada) by two EACVI CMR Level 3 certified operators (F.R. and N.A.), blinded to clinical and echocardiographic data. Ventricular volumes and function, as well as LV mass and atrial dimensions, were determined using balanced SSFP cine images as described previously.^14^

### Pulmonary transit time

**Figure 1:**
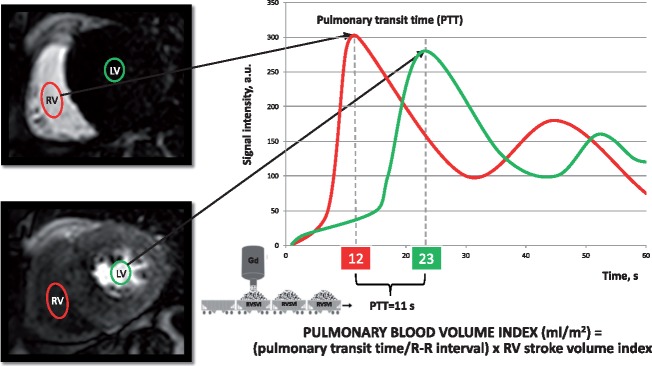
Pulmonary transit time and PBVI. SI/time curves of two regions of interest drawn in the RV cavity (red) and in the LV cavity (green), respectively. PTT is defined by the time interval between the peaks of the two SI/time curves. PBVI is obtained by the product between the RVSVI and the pulmonary transit time normalized by the R-R interval (see Methods section). Gd, gadolinium; LV, left ventricle; PBVI, pulmonary blood volume index; PTT, pulmonary transit time; RV, right ventricle; RVSVI, right ventricular stroke volume index; SI, signal intensity.

Pulmonary transit time (PTT) has been measured as the time interval required for a bolus of contrast agent to pass from the right ventricle to LV. First-pass perfusion imaging allows us to obtain an image for each heartbeat for 60 consecutive beats and can, therefore, be used to measure PTT. Briefly, in the basal slice of the first-pass perfusion imaging short-axis view stack, a region of interest was placed in the right ventricular (RV) cavity and copied in the same position for the entire stack of images. The average signal intensity (SI) of the region of interest was measured in every image and a SI/time curve was generated (*Figure 1*). A second SI/time curve was generated from another region of interest placed in the basal LV cavity. The PTT was measured as the peak-to-peak time interval between the two curves, both under resting and stress conditions. The number of cardiac cycles between the peaks of the two SI/time curves defines the number of pulmonary transit beats (PTB). Bazett’s formula was used to correct the PTT interval for the heart rate: PTTc (s) = PTT (s)/√R-R interval(s). In a preliminary study, we compared the measurement of PTT and PTB as onset-to-onset, peak-to-peak and half-width time interval of the wash-in phase of SI/heartbeat curves.^11^ We observed no significant differences among such methods, and we finally chose the peak-to-peak time interval as the most reproducible, yielding intra-class correlation coefficient values of 0.99 and 0.97 for intra-observer and inter-observer reliability, respectively.

### Pulmonary blood volume index

Resting pulmonary blood volume indexed to body surface area (PBVI, mL/m^2^) was measured by the product of PTB and the RV stroke volume index (RVSVI) (*Figure 1*). This is better understood if one considers PBVI as being analogous to a cargo train, with the number of wagons equivalent to the number of cardiac cycles for an intravenous bolus of gadolinium contrast to pass through the pulmonary circulation, and the content of each wagon equal to the RVSVI.

### Statistical analysis

We used the Kolmogorov–Smirnov test for normal distribution. Continuous variables were expressed as mean ± standard deviation or median and IQR, if not normally distributed. Categorical variables were expressed as frequency and percentage. The Student’s independent *t*-test, Mann–Whitney *U* test, analysis of variance (ANOVA), or Kruskal–Wallis test were used to compare continuous variables, while categorical variables were compared using the χ^2^ or Fisher’s exact test. Diagnostic accuracy of PBVI for the diagnosis of diastolic dysfunction was assessed by receiver operating characteristic (ROC) curve analysis. Uni- and multivariable logistic regression analyses were performed to determine predictors of diastolic dysfunction. The adjusted model was built by adding age, sex, and covariates found to be clinically relevant and/or statistically significant at univariate analysis. The significance level of all analyses was set at *P* < 0.05. Statistical analyses were performed using MedCalc for Windows, version 12.5 (MedCalc Software, Ostend, Belgium) and Wizard for Mac, version 1.7.8 (Boston, MA, USA).

## Results

Baseline characteristics of the study population are summarized in *Table 1*. All patients (mean age 58 ± 11 years; 83% of men) were diagnosed with HCM based on clinical, electrocardiographic, echocardiographic and CMR findings. Asymmetric septal (48%) and apical (30%) hypertrophy were the most frequent morphologic HCM phenotypes. Overall, 32 (46%) patients were in New York Heart Association (NYHA) Class I, 29 (42%) in Class II, and 8 (12%) in Class III. Of 23 patients with diastolic dysfunction and increased LAP, 6 (26%) showed a restrictive filling pattern (Grade III).

**Table 1:** Baseline characteristics of HCM outpatients by severity of diastolic dysfunction as assessed by transthoracic echocardiography

Characteristics	HCM (*n* = 69)	Increased LAP Grade II–III (*n* = 23)	Normal LAP Grade I (*n* = 46)	*P*-value
Age (years)	58 ± 11	57 ± 11	58 ± 11	NS^a^
Gender (male), *n* (%)	57 (83)	18 (78)	39 (85)	NS^b^
BMI (kg/m^2^), median (IQR)	29 (6)	29 (8)	28.5 (6)	NS^c^
BSA (m^2^), median (IQR)	1.99 (0.30)	2.00 (0.39)	1.98 (0.31)	NS^c^
Diabetes, *n* (%)	12 (17)	3 (13)	9 (19)	NS^b^
Active smoker, *n* (%)	29 (42)	10 (43)	19 (41)	NS^b^
Hypertension, *n* (%)	28 (40)	10 (43)	18 (39)	NS^b^
NYHA class, *n* (%)				
I	32 (46)	6 (26)	26 (57)	0.017^b^
II	29 (42)	10 (44)	19 (41)	NS^b^
III	8 (12)	7 (30)	1 (2)	<0.001^b^
IV	0 (0)	0 (0)	0 (0)	
Resting cardiac morphology and function by CMR imaging				
LVSVI (mL/m^2^), median (IQR)	62 (17)	61 (14)	50 (14)	<0.001^c^
LVEF (%)	73 ± 9	75 ± 8	72 ± 9	NS^a^
LVEDVI (mL/m^2^), median (IQR)	74 (22)	95 (23)	78 (14)	0.006^c^
LVESVI (mL/m^2^)	21 ± 10	21 ± 9	21 ± 10	NS^a^
RVSVI (mL/m^2^)	51 (15)	55 (12)	47 (12)	0.004^c^
RVEF (%)	68 ± 8	70 ± 9	67 ± 8	NS^a^
RVEDVI (mL/m^2^), median IQR	73 (15)	78 (14)	68 (19)	0.005^c^
RVESVI (mL/m^2^)	23 ± 10	24 ± 8	23 ± 11	NS^a^
LA area index (cm^2^/m^2^)	14 ± 4	15 ± 4	14 ± 4	NS^a^
LA volume index (mL/m^2^)	56 ± 12	69 ± 13	50 ± 12	<0.001^a^
AP-LA diameter (mm)	40 ± 8	40 ± 12	40 ± 7	NS^a^
Maximal wall thickness (mm)	18 ± 5	18 ± 4	18 ± 5	NS^a^
LV mass index (g/m^2^)	91 ± 25	93 ± 32	90 ± 22	NS^a^
SAM, *n* (%)	20 (30)	10 (43)	10 (22)	NS^b^
LVOTO, *n* (%)	11 (16)	4 (17)	7 (15)	NS^b^
MR,^d^*n* (%)	8 (12)	6 (26)	2 (4)	0.015^b^
LGE, *n* (%)	48 (70)	19 (83)	29 (63)	NS^b^
Adenosine-induced perfusion defects, *n* (%)	52 (75)	19 (82)	33 (72)	NS^b^
Heart rate (bpm), rest	68 ± 11	67 ± 10	68 ± 11	NS^a^
Heart rate (bpm), stress	84 ± 13	81 ± 4.5	87 ± 5.8	NS^a^
Diastolic function assessment				
PTT (s)	6.7 ± 1.7	7.2 ± 1.5	6.4 ± 1.7	0.042^a^
PTTc (s)	6.8 ± 1.6	7.7 ± 1.4	6.5 ± 1.6	0.014^a^
PTTc stress (s)	7.5 ± 2	7.7 ± 2.1	7.4 ± 2.1	NS^a^
PTB (bpm)	7.4 ± 1.8	8.0 ± 1.5	7.1 ± 1.8	0.047^a^
PBVI (mL/m^2^)	361 ± 124	463 ± 127	310 ± 86	<0.001^a^
PFRE (mL/s)	641 ± 201	666 ± 189	628 ± 207	NS^a^
PFRE/BSA (mL/m^2^)	333 ± 101	352 ± 107	323 ± 98	NS^c^
PFRE/EDV (/s)	4.5 ± 1.5	4.4 ± 1.2	4.7 ± 1.6	NS^c^
PFRA (mL/s)	505 ± 196	563 ± 243	477 ± 165	NS^a^
PFRA/BSA (mL/m^2^)	261 ± 91	293 ± 107	245 ± 79	NS^c^
PFRA/EDV (/s)	3.5 ± 1.1	3.7 ± 1.2	3.5 ± 1.1	NS^c^
PFRE/PFRA	1.4 ± 0.4	1.3 ± 0.4	1.4 ± 0.5	NS^c^
sPAP (mmHg),^e^ median (IQR)	29 (13)	37 (16)	27 (9)	0.003^c^
E wave (cm/s),^e^ median (IQR)	70 (26)	78 (17)	62 (26)	0.009^c^
*E*/*A,*^e^ median (IQR)	1.2 (0.5)	1.2 (0.4)	1.1 (0.5)	NS^c^
LAVI (mL/m^2^),^e^ median (IQR)	49 (26)	68 (45)	47 (14)	0.011^c^
*E*/*E*',^e^ median (IQR)	12 (5)	15 (3.5)	11 (4)	<0.001^c^

Data are presented as mean ± SD unless otherwise indicated.

AP, anteroposterior; BMI, body mass index; BSA, body surface area; EDV, end-diastolic volume; HCM, hypertrophic cardiomyopathy; IQR, interquartile range; LA, left atrium; LAP, left atrial pressure; LAVI, left atrial volume index; LGE, late gadolinium enhancement; LV, left ventricle; LVEDVI, left ventricular end-diastolic volume index; LVEF, left ventricular ejection fraction; LVESVI, left ventricular end-systolic volume index; MR, mitral regurgitation; NS, not significant; PBVI, pulmonary blood volume index; PFRA, atrial (active) peak filling rate; PFRE, early (passive) peak filling rate; PTB, pulmonary transit beats; PTT, pulmonary transit time; PTTc, pulmonary transit time corrected by Bazett's formula; RA, right atrium; RVEDVI, right ventricular end-diastolic volume index; RVEF, right ventricular ejection fraction; RVESVI, right ventricular end-systolic volume index; SAM, systolic anterior movement; SD, standard deviation; sPAP, systolic pulmonary artery pressure.

a The Student's *t*-test for unpaired data.

b The χ^2^ test.

c The Mann–Whitney *U* test.

d At least moderate.

e Assessed by transthoracic echocardiography.

Compared with patients without elevated LAP, patients with increased LAP showed significantly larger biventricular end-diastolic volumes, stroke volumes and left atrium volume index, longer PTTc, larger PBVI, and more severe mitral regurgitation (*Table 1*).

Presence and extent of myocardial fibrosis, adenosine inducible perfusion defects, and prevalence of systolic anterior movement and LV outflow obstruction were similar between the two groups (*P* = not significant).

Compared with resting measurement, PTTc significantly increased during adenosine stress in patients with normal LAP (*P* = 0.045), remaining unchanged in the subgroup of patients with elevated LAP.

**Figure 2:**
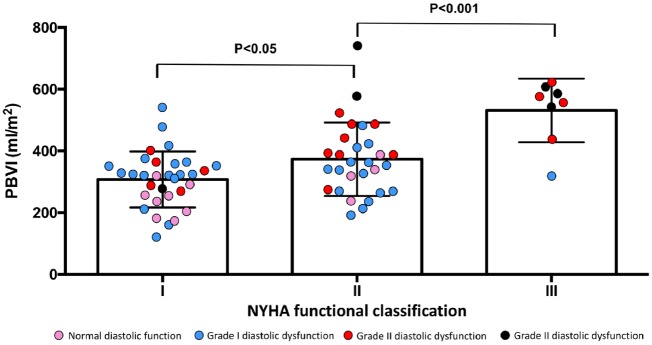
PBVI by NYHA functional class status. NYHA, New York Heart Association; PBVI, pulmonary blood volume index.

**Figure 3:**
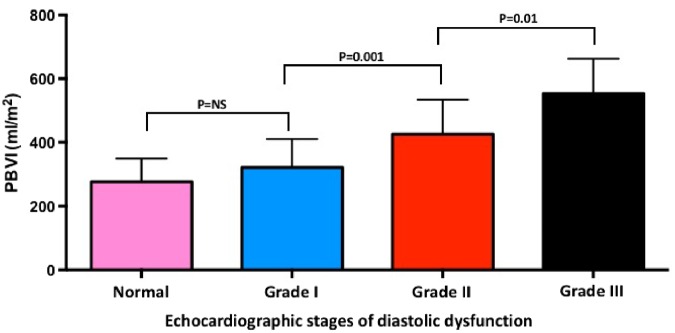
PBVI and echocardiographic stages of diastolic dysfunction. PBVI, pulmonary blood volume index.

PBVI increased progressively with worsening NYHA functional class (*Figure 2*) and echocardiographic stages of diastolic dysfunction (ANOVA; *P* < 0.001 for both) (*Figure 3*). In the subgroup of asymptomatic or mildly symptomatic patients (NYHA functional Class I or II), patients with increased LAP showed higher PBVI compared with patients with normal LAP (PBVI 419 ± 125 vs. 308 ± 88 mL/m^2^; *P* < 0.0001).

**Figure 4:**
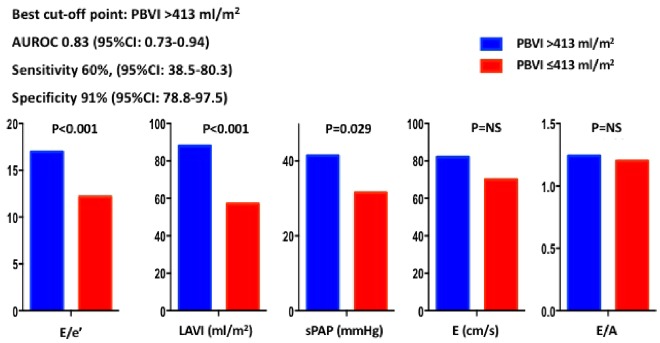
Diagnostic accuracy of PBVI for the diagnosis of diastolic dysfunction with increased mean left atrial pressure. AUROC, area under the receiver operating characteristic curve; CI, confidence interval; PBVI, pulmonary blood volume index.

**Figure 5:**
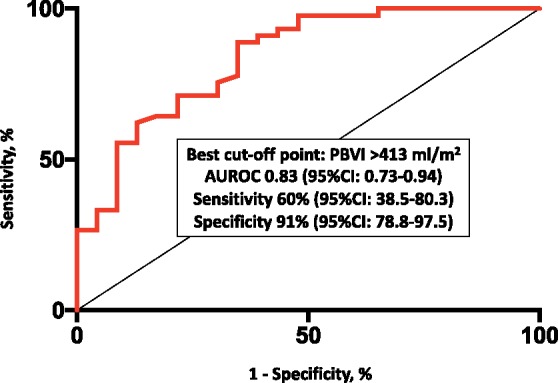
Echocardiographic parameters of diastolic function in patients with higher and lower PBVI. Patients with PBVI >413 mL/m^2^ (corresponding to the best cut-off point derived from receiver operating characteristic curve analysis) had significantly higher values of *E*/*e*’, LAVI, and sPAP than the other subgroup of patients with PBVI ≤413 mL/m^2^. AUROC, area under the ROC curve; *E*/*A*, mitral valve *E* velocity divided by A-wave velocity; *E*/*e*’, mitral valve *E* velocity divided by mitral annular *e*’ velocity; PBVI, pulmonary blood volume index; LAVI, left atrial volume index; sPAP, systolic pulmonary artery pressure.

PBVI yielded overall good diagnostic accuracy for diastolic dysfunction [area under the ROC curve 0.83, 95% confidence interval (CI) 0.73–0.94], with the best cut-off point corresponding to 413 mL/m^2^ and leading to a sensitivity of 60% (95% CI 38.5–80.3) and a specificity of 91% (95% CI 78.8–97.5) (*Figure 4*). *E*/*e*’, LA volume index, and sPAP were significantly higher in patients with PBVI >413 mL/m^2^ as compared to those below this threshold (*Figure 5*). PBVI was significantly associated with several echocardiographic indices of diastolic dysfunction and showed significant positive correlation with tissue-Doppler *E*/*e*’ ratio (*r* = 0.275, *P* = 0.026), sPAP (*r* = 0.284, *P* = 0.02), and LA volume index (*r* = 0.506, *P* < 0.001). On multivariate logistic regression analysis, PBVI was an independent predictor of LV diastolic dysfunction with elevated LAP (*Table 2*).

**Table 2:** Univariate and multivariate logistic regression analysis of CMR predictors of haemodynamic congestion

Characteristics	Univariate		Multivariate^a^	
	Odds ratio (95% CI)	*P*-value	Odds ratio (95% CI)	*P*-value
Age (years)	1.01 (0.95–1.04)	0.748	0.98 (0.88–1.09)	0.679
Male sex	0.65 (0.18–2.32)	0.502	0.04 (0.0–5.95)	0.204
NYHA class	3.96 (1.67–9.39)	0.002	0.95 (0.16–5.52)	0.305
LVEDVI (mL/m^2^)	1.04 (1.01–1.07)	0.022	0.7 (0.32–1.53)	0.366
LVSVI (mL/m^2^)	1.09 (1.03–1.14)	0.002	1.63 (0.54–4.9)	0.386
LVEF (%)	1.05 (0.98–1.12)	0.144	0.66 (0.26–1.66)	0.375
RVEDVI (mL/m^2^)	1.06 (1.01–1.11)	0.043	0.65 (0.37–1.16)	0.146
RVSVI (mL/m^2^)	1.13 (1.03–1.23)	0.008	2.19 (0.85–5.59)	0.103
RVEF (%)	1.04 (0.96–1.12)	0.327	0.58 (0.29–1.14)	0.115
MR^b^ (binary)	2.92 (1.02–8.34)	0.046	0.27 (0.25–6.34)	0.770
LGE (binary)	2.88 (0.84–9.92)	0.093	4.0 (0.24–65.44)	0.332
Adenosine inducible perfusion defects (binary)	1.93 (0.55–6.78)	0.305	0.35 (0.02–7.62)	0.507
LV mass index^c^ (g/m^2^)	1.01 (0.84–1.2)	0.932	0.86 (0.55–1.33)	0.511
PBVI^c^ (mL/m^2^)	1.77 (1.34–2.33)	<0.001	1.97 (1.06–3.68)	0.032

CI, confidence interval; LGE, late gadolinium enhancement; LVEDVI, left ventricular end-diastolic volume index; LVEF, left ventricular ejection fraction; NYHA, New York Heart Association; PBVI, pulmonary blood volume index.

a Adjusted for all covariates (CI, LGE, LVEDVI, LVEF, NYHA, and PBVI.) listed in the tables.

b At least moderate.

c Per 10% increase.

## Discussion

In the present study, we used first-pass perfusion CMR imaging to estimate pulmonary blood volume and transit time in patients with HCM. The major findings of this study were that: (i) increases in PBVI yield good diagnostic accuracy for the detection of diastolic dysfunction with elevated LAP (as defined by echocardiography), providing excellent specificity at the best cut-off level of 413 mL/m^2^; (ii) HCM patients with Grade II or III diastolic dysfunction also showed significantly larger PBVI and longer PTTc as compared to patients with normal LAP; and (iii) the magnitude of PBVI correlates with the severity of diastolic dysfunction and worsening functional capacity.

The symptoms in patients with HCM largely result from elevated mean LAP (‘backward’ HF).^1^ However, in many cases backward and forward failure coexist in a complex interplay of pathophysiological mechanisms, including coronary microvascular dysfunction with reduced arteriolar density and structural abnormalities of intramural coronary arterioles, replacement fibrosis, myocyte disarray, mitral regurgitation associated with systolic anterior movement, and LV outflow tract obstruction as well as elevated LV end-diastolic pressure, eventually leading to the elevation of pulmonary capillary hydrostatic pressure (or haemodynamic congestion) and, at an advanced stage, to overt pulmonary congestion.^15–18^

Since haemodynamic derangements usually precede clinical symptoms, the ability to detect and grade haemodynamic congestion from non-invasive measurements is a highly desirable though unmet clinical need.^19^ Echocardiography is the recommended non-invasive imaging modality for the evaluation of LV diastolic function, but may be sometimes misleading, with key indices yielding discrepant information, especially in HCM,^20^ and the use of many parameters is perceived too complex and not practical to perform and repeat on every patient.^8^ Furthermore, diagnostic accuracy of the 2009 and 2016 echocardiographic grading algorithms for the diagnosis of LV diastolic dysfunction has been recently questioned by the results of the multicentre EACVI Euro-Filling study.^21^

CMR is the gold-standard imaging technique for the non-invasive quantification of blood flow and volumes^22^ and has been increasingly recognized as a valuable complement to TTE for the clinical evaluation of HCM, providing a number of unique strengths which make it particularly well suited to detailed characterization of the HCM phenotype, and therefore, an important aid for diagnosis and potentially prognosis.^23–26^

Furthermore, comprehensive CMR imaging protocols have been advocated to improve diagnosis and targeted management of HF with preserved LV ejection fraction.^27^

While a non-invasive gold standard for the quantification of diastolic function in HCM is still an unmet clinical need,^28^ CMR has been proposed as an alternative non-invasive modality for the evaluation of LV diastolic function, although the relatively low temporal resolution—i.e. 20–30 ms—limited its clinical application so far.^29^^,^^30^ More recently, PBVI by CMR has proved to be helpful to quantitatively determine haemodynamic congestion—meaning a greater volume of blood in the pulmonary circulation leading to higher hydrostatic pressure—in HF outpatients with reduced ejection fraction^11^ and adults with repaired congenital heart disease.^31^

In this study, we confirmed previous findings^11^^,^^32^ and reported a positive relationship between PBVI and echocardiographic stages of diastolic dysfunction in a cohort of both asymptomatic and symptomatic HCM patients. Furthermore, increasing values of PBVI were significantly associated with worsening functional status, i.e. higher NYHA functional class and lower exercise time duration.

Overall, PBVI might be considered as a quantitative and reproducible biomarker of haemodynamic congestion—a well-recognized unmet clinical need^19^—and might represent a specific tool to unmask poor haemodynamic reserve, eventually useful for the clarification of the pathophysiology underlying impaired functional capacity and HF symptoms in HCM patients. Assessment of resting PBVI and transit time kinetics may compliment functional analysis and tissue characterization and can be ideally performed on a routine basis without affecting total scan time duration.

After adenosine infusion, PTTc significantly increased in patients with better diastolic function, while it remained unchanged in patients with diastolic dysfunction and elevated LAP. We know that similar to the systemic and coronary circulation, the pulmonary arteries dilate in response to purine nucleoside adenosine which has a direct endothelium-independent effect on the A2b receptor in vascular smooth muscle.^33^ We may argue that reduced pulmonary resistance in response to the endothelium-independent vasodilatory effect of adenosine would lead to an increased transit time of the contrast agent (PTT = PBV/cardiac output) due to the distention of the pulmonary vascular bed and recruitment of the pulmonary microcirculation, overall expanding the pulmonary capillary surface area and volume.^34^ Conversely, pulmonary vasodilatory reserve may have been exhausted in our HCM patients with elevated LAP. However, other authors would consider constant PTTc at rest and during stress in keeping with an intact vasoreactive response to accommodate increased cardiac output during adenosine infusion.^33^ A number of factors may act on endothelium-independent function of the pulmonary vasculature and accuracy of PTT measurement, namely active cigarette smoking, which has been associated with impaired vasoreactivity during CMR adenosine stress testing, adenosine dosing and delay between stress and rest perfusion imaging acquisition. Nevertheless, the comparison of pulmonary blood volume variation over the cardiac cycle, as previously described,^12^^,^^35^ at rest and during adenosine stress would best explain the differences in central transit time kinetics observed between patients with increased and normal LAP.

Further studies are warranted in order to: (i) assess the clinical utility and prognostic value of serial PBVI analysis in HCM population as well as in other clinical settings; (ii) investigate the association between PBVI and serum levels of natriuretic peptides; (iii) investigate the relationship between symptoms and PBVI before and after structural interventions, i.e. alcohol septal ablation; (iv) test the effect of adenosine—and/or exercise stress CMR—on pulmonary vascular function and right heart-pulmonary circulation unit; (v) test the potential usefulness PBVI in the assessment of HF with preserved LV ejection fraction, specifically in the subgroup in which diastolic function assessment according to the current guidelines is normal or indeterminate; and (vi) test the diagnostic accuracy of PBVI with established gold-standard invasive techniques for the measurement of pulmonary artery wedge pressure and LV end-diastolic pressure.

Finally, from a wider perspective, adenosine stress CMR imaging might represent a unique tool for simultaneous assessment of myocardial ischaemia—also able to distinguish between obstructive epicardial CAD and microvascular coronary dysfunction^36^—and diastolic function reserve, which are both involved in a vicious cycle harbinger of future risk of HF with preserved ejection fraction hospitalization.^37^

### Limitations

A few limitations should be addressed. Firstly, due to the retrospective design, the referral to stress perfusion CMR imaging may have introduced a selection bias of patients with higher burden of symptoms, however, most patients presented with NYHA functional III or better. Secondly, right ventricular stroke volume was calculated as the difference between end-diastolic and end-systolic volume. For the purpose of this study, measurement of forward flow by phase-contrast velocity mapping would have been more precise; however, exclusion of patients with moderate or severe tricuspid regurgitation attenuated possible inaccuracy. Thirdly, we could not exclude coexisting coronary artery disease as a possible mechanism underlying diastolic dysfunction in all patients, however, this was out of the main scope of our analysis. Fourthly, because of the limited sample size, the multivariable analysis is likely to be underpowered to derive a robust predictive model and our findings should only be intended as hypothesis-generating. Fifthly, the accuracy of measuring PBVI may be limited due to the uncertain relationship between the bolus concentration of contrast material and the SI on CMR images used for PTT measurements. Sixthly, we did not obtain cardiac volumes during stress and could not assess changes in PBVI and cardiac output after adenosine administration. Furthermore, invasive haemodynamic measurements derived from right and/or left heart catheterization were not available in the current study, however it has been recently showed that PTT prolongation was significantly associated with haemodynamic abnormalities at invasive haemodynamic testing by right and left heart catheterization such as elevated pulmonary capillary wedge pressure, LV end-diastolic pressure, reduced cardiac index, and oxygen saturation, of which increased pulmonary capillary wedge pressure demonstrated the strongest association.^10^ Finally, serum levels of natriuretic peptides, cardiopulmonary exercise testing data, and T1 mapping indices were not routinely available in our series.

## Conclusions

PBVI analysis is a promising application for assessment of haemodynamic congestion which may effectively compliment the functional assessment and tissue characterization by CMR in patients with HCM. Future prospective studies should investigate the prognostic role and the clinical utility of PBVI in targeted management of patients with HCM, fostering the wider objective of imaging-guided precision medicine in HF.
